# Modularized Perturbation of Alternative Splicing Across Human Cancers

**DOI:** 10.3389/fgene.2019.00246

**Published:** 2019-04-03

**Authors:** Yabing Du, Shoumiao Li, Ranran Du, Ni Shi, Seiji Arai, Sai Chen, Aijie Wang, Yu Zhang, Zhaoyuan Fang, Tengfei Zhang, Wang Ma

**Affiliations:** ^1^Department of Oncology, The First Affiliated Hospital of Zhengzhou University, Zhengzhou, China; ^2^Department of Surgery, Anyang Tumor Hospital, Anyang, China; ^3^Institute of Medical Information, Chinese Academy of Medical Sciences, Peking Union Medical College, Beijing, China; ^4^Comprehensive Cancer Center, Ohio State University, Columbus, OH, United States; ^5^Department of Hematology and Oncology, Beth Israel Deaconess Medical Center, Harvard Medical School, Boston, MA, United States; ^6^Department of Urology, Gunma University Graduate School of Medicine, Maebashi, Japan; ^7^Department of Clinical Medicine, Xinxiang Medical University, Xinxiang, China; ^8^Bioinformatics Group, Medcurius Co., Zhejiang, China; ^9^Shanghai Institutes for Biological Sciences, Chinese Academy of Science, Shanghai, China; ^10^Medical College, Henan Polytechnic University, Jiaozuo, China

**Keywords:** alternative splicing, splicing network, splicing modules, cancer splicing, prognosis

## Abstract

Splicing perturbation in cancers contribute to different aspects of cancer cell progression. However, the complete functional impact of cancer-associated splicing have not been fully characterized. Comprehensive large-scale studies are essential to unravel the dominant patterns of cancer-associated splicing. Here we analyzed the genome-wide splicing data in 16 cancer types with normal samples, identified differential splicing events in each cancer type. Then we took a network-based and modularized approach to reconstruct cancer-associated splicing networks, determine the module structures, and evaluate their prognosis relevance. This approach in total identified 51 splicing modules, among which 10/51 modules are related to patient survival, 8/51 are related to progression-free interval, and 5/51 are significant in both. Most of the 51 modules show significant enrichment of important biological functions, such as stem cell proliferation, cell cycle, cell growth, DNA repair, receptor or kinase signaling, and VEGF vessel development. Module-based clustering grouped cancer types according to their tissue-of-origins, consistent with previous pan-cancer studies based on integrative clustering. Interestingly, 13/51 modules are highly common across different cancer types, suggesting the existence of pan-cancer splicing perturbations. Together, modularized perturbation of splicing represents an functionally important and common mechanism across cancer types.

## Introduction

Newly transcribed messenger RNAs undergo processing steps such as capping, splicing and polyadenylation to derive mature RNAs for export and translation (Hocine et al., 2010). The splicing process, as accomplished by the spliceosome machine, can produce multiple alternative products, which is a well-known phenomena called alternative splicing (AS) (Lee and Rio, 2015). Since its first discovery in 1977, many classical studies have characterized its widespread participation in biological processes such as cell proliferation, apoptosis, angiogenesis, neuronal functions, and transcriptional regulation (Kelemen et al., 2013). Deregulation of AS also contributes to human diseases and various aspects of cancer development (David and Manley, 2010; Scotti and Swanson, 2016).

To systematically characterize the extensive cancer-associated AS perturbations, it is essential to design effective analytic strategies suitable for the ever-growing cancer genomic datasets, largely from projects such as The Cancer Genome Atlas (TCGA). Several approaches have already been taken previously. One of the most popular approaches is the event-driven approach, which aimed to detect individual events that are correlated with cancer or prognosis (Danan-Gotthold et al., 2015; Dvinge and Bradley, 2015; Shen et al., 2016). A second approach focuses on the splicing machinery side and tries to determine the deregulation of splicing factors in tumors (Sebestyén et al., 2016; Sveen et al., 2016; Seiler et al., 2018). Since these approaches emphasized different aspects of the AS perturbations, several recent studies have been linking the splicing factors and events together to identify AS deregulation and the corresponding functional impacts (Li et al., 2017; Kahles et al., 2018). However, this approach may oversee the vast majority of perturbed splicing events that are not easily explained by the few known regulatory factors (Li et al., 2017). Moreover, these studies essentially relied on single-event analysis, and have missed the inter-event linkages which could be equally important to fully understand cancer-specific AS perturbations. To complement these analyses, a fully network-based approach is needed to capture the concurrent perturbation patterns of cancer-associated AS. In addition, such an approach might also discover more robust AS patterns in one or multiple cancer types.

We carried out an extensive analysis of AS events and their interactions in different cancer types. For each cancer type, a network of cancer-associated events is reconstructed. To uncover the potential modularized control in these splicing networks, a random walk-based community identification algorithm is employed. These analyses have revealed representative splicing modules in each type of cancers, and a number of them are prognosis-relevant and involved in cancer-related functional processes. Finally, our work supports the unique value of an splicing network-based approach in understanding cancer splicing deregulation.

## Materials and Methods

### Data Sets and Processing

Splicing data have been downloaded from the TCGASpliceSeq database (Ryan et al., 2016). Clinical information is from the GDC TCGA project. Splicing events that failed to be quantified >10% in normal samples or >1% in cancer samples were filtered without further use. Cancer samples with >0.1% missing data were also removed. The remaining missing values were imputed with the Bioconductor impute package.

### Network Reconstruction and Module Identification

For each cancer type, Pearson correlation coefficients among splicing events were computed between each pair of the differentially spliced events (Wilcoxon signed-rank test FDR <0.1, |delta PSI|>0.1), and were used as edge weights in the reconstructed undirected graph. The Pons-Lapaty random walk algorithm (step = 4) was used to partition the weighted graph. The identified modules from each cancer type were named according to the order of module sizes (from larger to smaller). So M1 is always at least as large as M2, and M2 at least as large as M3, etc.

### Overall and Progression-Free Survival Analysis

Module scores are averaged from all splicing events in each sample, with normal sample PSI values used as references and subtracted. Thus, the score measures how strong the module is perturbed in one cancer sample. To ensure robustness, both the average and median scores have been calculated. Overall survival (OS) and progression-free intervals (PFI) are respectively categorized for testing with module scores. The Kaplan-Meier survival curves are fitted and compared between samples with a higher vs. a lower module score using the Log-rank test. Hazard ratios and confidence intervals are estimated from the Cox proportional regression model. In total, 13 modules were found to be significantly correlated to either OS (10 modules) or PFI (8 modules). Of these, 5 modules were commonly significant in both OS and PFI. In addition, 2 Lung squamous cell carcinoma (LUSC) modules were nearly significant in OS and also included as candidates. Therefore, 15 modules were retained after filtering with OS and PFI analyses.

### Functional Enrichment Analyses

Gene ontology (GO) enrichment was used to assess the functional properties of each module. The enrichment was determined by the Fisher's exact test method. For all significant GO terms, careful manual inspection and curation were performed to find the most relevant and biologically important functions, which is often a subset of the significant terms.

## Results

### Splicing Network-Based Flowchart for Identifying Prognosis-Relevant Splicing Modules

**Figure 1:**
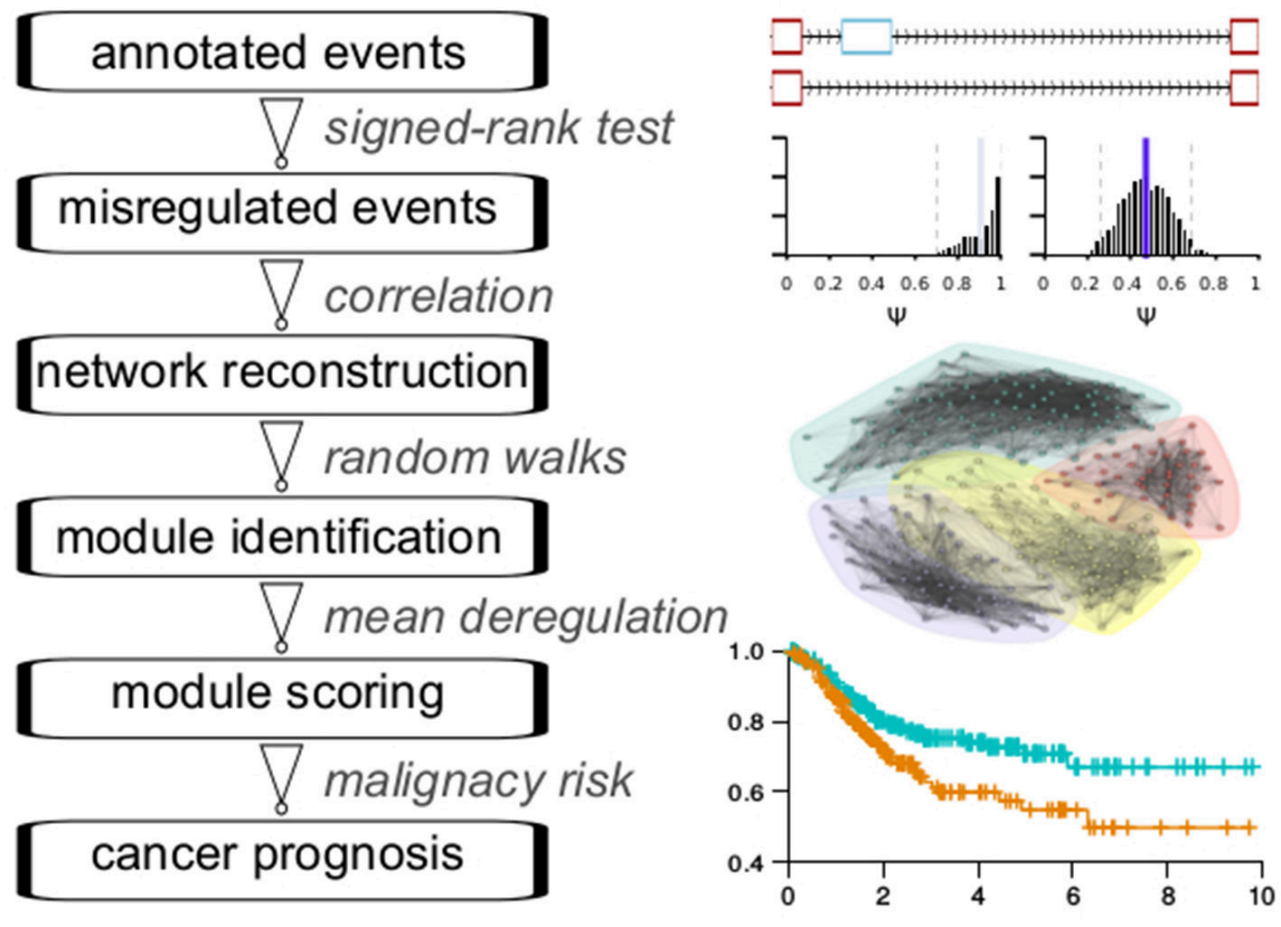
Flowchart of analyses.

The main analytic flowchart consist of six steps (Figure 1): (1) Collection of annotated events from the TCGASpliceSeq database across 33 cancer types. The splicing classes included are exon skipping (ES), retention of introns (RI), alternative donor (AD), alternative acceptor (AA), mutually exclusive exons (ME), alternative terminator (AT). The numbers of quantified events were found in at least 99% of the samples in each cancer type range from 21129 to 43937 across the cancer types. (2) Differential splicing events between cancer samples and adjacent normal samples were identified for 16 cancer types with at least 10 normal samples. The Wilcoxon signed-rank test was used for testing. The number of differential events obtained ranges from 228 in ESCA to 1133 in LUSC. (3) Reconstruction of splicing network for each cancer type, with Pearson correlation coefficient-based similarity linkages. Pearson and Kendall correlation coefficients showed a good consistency in subsequent community detection, confirming the reliability of this procedure (Figure S1). (4) Network module identification with the Pons-Lapaty algorithm which is based on random walks in 3–5 steps to measure vertex distances for hierarchical clustering and subsequent modularity-optimized graph partition (Pons and Latapy, 2005). (5) Modules are then scored with the averaged splicing deregulation between each cancer sample and the normal samples, which provide a reasonable quantification of module-level perturbation across cancer samples. (6) Prognosis analyses for each module and its corresponding cancer type. Figure 1 shows a schematic diagram of these steps.

### Cancer Splicing Networks and Modules for TCGA Cancer Types

**Figure 2:**
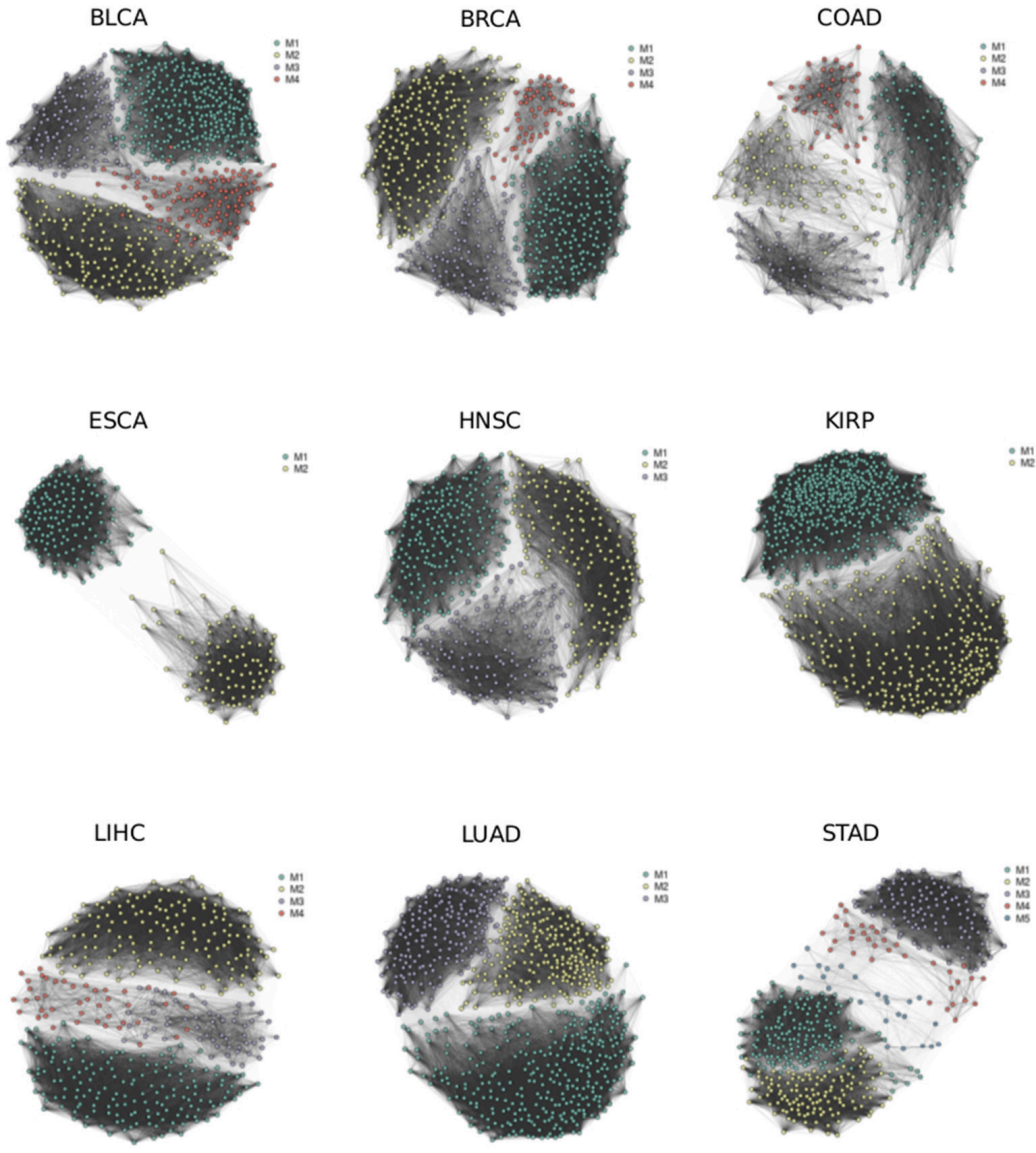
Splicing networks for different cancer types. Modules identified by random walks are highlighted in different colors.

We reconstructed splicing networks for each cancer type with at least 10 normal samples on differential events. There are 16 cancer types that bypass the above criteria, namely: Bladder urothelial carcinoma (BLCA), Breast invasive carcinoma (BRCA), Colon adenocarcinoma (COAD), Esophageal carcinoma (ESCA), Head and Neck squamous cell carcinoma (HNSC), Kidney Chromophobe (KICH), Kidney renal clear cell carcinoma (KIRC), Kidney renal papillary cell carcinoma (KIRP), Liver hepatocellular carcinoma (LIHC), Lung adenocarcinoma (LUAD), Lung squamous cell carcinoma (LUSC), Prostate adenocarcinoma (PRAD), Rectum adenocarcinoma (READ), Stomach adenocarcinoma (STAD), Thyroid carcinoma (THCA), and Uterine Corpus Endometrial Carcinoma (UCEC). The number of modules identified with the Pons-Lapaty algorithm varied between 2 and 5 for these cancer types. The visualized inspection revealed quite clear network partitions (Figure 2). In total, 51 modules were identified, and the number of events and genes in each module can be found in Table 1 and Table S1. For example, the KIRC_M1 module consisted of 710 events in 630 genes, with 41 AA events, 36 AD events, 229 AT events, 210 ES events, 3 ME events, and 191 RI events.

**Table 1:** A list of 51 cancer splicing modules.

**Module**	**Genes**	**Events**	**AA**	**AD**	**AT**	**ES**	**ME**	**RI**
BLCA_M1	302	332	22	19	130	73	0	88
BLCA_M2	188	191	4	0	152	30	1	4
BLCA_M3	118	119	8	7	91	12	0	1
BLCA_M4	104	111	3	4	73	26	1	4
BRCA_M1	239	257	14	8	137	73	1	24
BRCA_M2	216	221	8	8	158	37	2	8
BRCA_M3	107	121	6	5	54	40	1	15
BRCA_M4	51	52	2	2	25	19	1	3
COAD_M1	91	94	2	0	67	15	0	10
COAD_M2	57	66	2	1	48	8	1	6
COAD_M3	56	56	1	4	49	1	0	1
COAD_M4	42	43	2	3	16	6	0	16
ESCA_M1	132	141	5	13	60	32	0	31
ESCA_M2	86	87	1	8	64	12	1	1
HNSC_M1	200	220	14	9	93	53	1	50
HNSC_M2	159	165	3	4	126	28	0	4
HNSC_M3	116	127	7	2	84	29	0	5
KICH_M1	449	490	23	23	224	125	1	94
KICH_M2	363	383	10	19	232	90	5	27
KICH_M3	272	290	7	17	164	78	2	22
KIRC_M1	630	710	41	36	229	210	3	191
KIRC_M2	279	286	1	3	243	30	5	4
KIRC_M3	104	137	8	5	80	38	2	4
KIRP_M1	442	483	34	29	189	110	0	121
KIRP_M2	283	324	6	8	262	33	2	13
LIHC_M1	163	169	5	7	105	28	1	23
LIHC_M2	148	154	6	4	118	21	0	5
LIHC_M3	49	53	2	4	30	8	0	9
LIHC_M4	42	43	2	1	36	3	0	1
LUAD_M1	305	321	11	4	212	79	4	11
LUAD_M2	217	230	13	21	71	64	0	61
LUAD_M3	192	196	7	7	132	42	3	5
LUSC_M1	526	574	16	16	312	208	5	17
LUSC_M2	397	428	20	38	235	113	6	16
LUSC_M3	281	333	19	24	128	81	1	80
PRAD_M1	202	220	11	9	82	51	0	67
PRAD_M2	105	105	1	1	80	19	0	4
PRAD_M3	8	11	0	0	11	0	0	0
READ_M1	186	199	11	17	106	31	1	33
READ_M2	132	154	10	7	66	29	1	41
READ_M3	122	131	3	1	67	52	0	8
STAD_M1	141	164	11	13	37	41	0	62
STAD_M2	123	130	9	10	51	51	2	7
STAD_M3	108	112	1	3	71	35	1	1
STAD_M4	34	34	0	0	25	8	0	1
STAD_M5	17	24	1	1	16	4	0	2
THCA_M1	157	168	0	2	128	22	0	16
THCA_M2	149	151	4	2	114	17	0	14
UCEC_M1	292	302	7	5	244	31	2	13
UCEC_M2	206	210	4	4	172	14	0	16
UCEC_M3	117	121	4	7	65	27	1	17

*AA, alternative acceptor; AD, alternative donor; AT, alternative terminator; ES, exon skipping; ME, mutually exclusive exons; RI, retention of introns. For cancer type abbreviations, see text*.

### Overall and Progression-Free Survival Analyses for Cancer Splicing Modules

**Figure 3:**
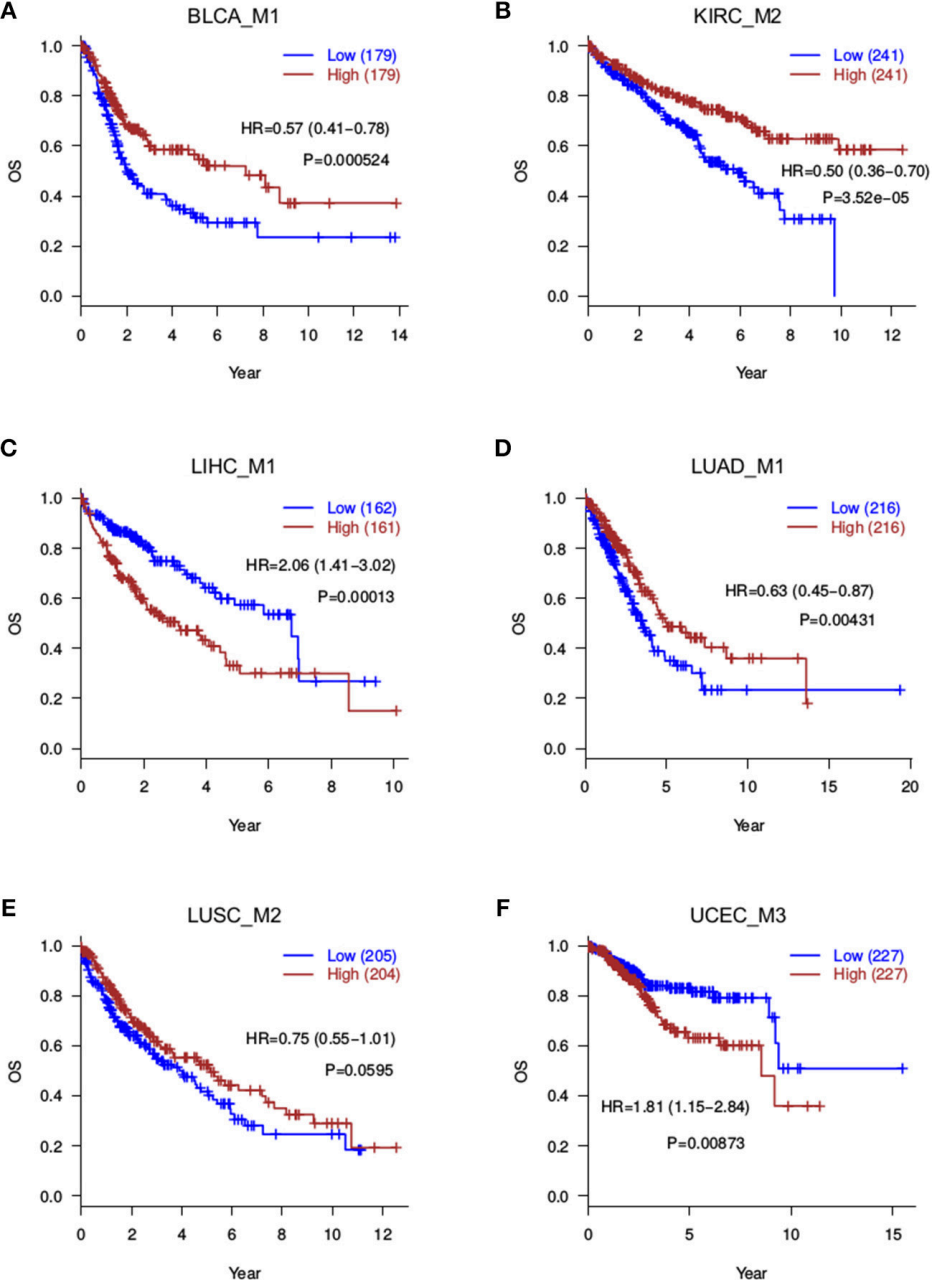
Overall survival analyses for splicing modules. Red curves are with a high module score, and blue curves with a low module score. **(A)** BLCA_M1; **(B)** KIRC_M2; **(C)** LIHC_M1; **(D)** LUAD_M1; **(E)** LUSC_M2; **(F)** UCEC_M3.

Since the motivation of this study is to discover prognosis-related splicing modules, we quantified module scores in each cancer sample and test associations between module scores and patient survival. Both the average score and median score were computed and assessed for prognosis correlation, and a very good consistency was found (Figure S2), indicating robustness of the module scoring procedure. At a 0.05 significance level, Log-rank tests identified 10 prognosis-related modules: BLCA_M1, BLCA_M2, KIRC_M1, KIRC_M2, LIHC_M1, LIHC_M2, LUAD_M1, LUAD_M3, PRAD_M1, and UCEC_M3 (Table 2, Figure 3). Two additional modules, the LUSC_M2 (*P* = 0.0595, HR = 0.75 with a confidence interval 0.55–1.01) and the LUSC_M3 (*P* = 0.099, HR = 0.77 with a confidence interval 0.57–1.05), are close to the significance level, and therefore are still likely to be potential prognosis biomarkers (Figure 3E). Notably, LUSC_M2 contains a ME event on the known LUSC amplification gene FGFR1 (exons 12.1:12.2 vs. exon 13), which could be functionally important in LUSC (Weiss et al., 2010; Heist et al., 2012).

**Table 2:** Cancer splicing modules correlated with prognosis.

**Module**	**OS_HR**	**OS_P**	**PFS_HR**	**PFS_P**	**Function**
BLCA_M1	0.57 (0.41–0.78)	0.00052	0.67 (0.48–0.91)	0.011	Stem cell proliferation; EMT
BLCA_M2	1.78 (1.29–2.48)	0.00044	1.42 (1.04–1.95)	0.027	Microtubule bundle; actin filament polymerization
BLCA_M4	0.75 (0.54–1.03)	0.076	0.70 (0.51–0.97)	0.029	Cell junction; Rac and Ras signaling
KIRC_M1	2.04 (1.46–2.84)	2E−05	1.24 (0.89–1.74)	0.2	mRNA splicing and export; transcription termination
KIRC_M2	0.50 (0.36–0.70)	3.5E−05	0.77 (0.55–1.08)	0.12	Drug metabolism; PI3K signaling; amino acid metabolism
LIHC_M1	2.06 (1.41–3.02)	0.00013	1.32 (0.96–1.82)	0.085	ERK1/2 signaling; organ growth; PI3K signaling
LIHC_M2	0.50 (0.34–0.73)	0.00023	0.79 (0.57–1.08)	0.14	ERK1/2 signaling; stem cell proliferation; embryonic epithelium
LUAD_M1	0.63 (0.45–0.87)	0.0043	0.74 (0.55–1.01)	0.054	PKC signaling; VEGF and lymph vessel development
LUAD_M3	1.86 (1.34–2.58)	0.00018	1.42 (1.05–1.93)	0.023	Mitosis; double-strand break repair
LUSC_M2	0.75 (0.55–1.01)	0.059	0.80 (0.56–1.15)	0.22	Phospholipase activity; formation of primary germ layer
LUSC_M3	0.77 (0.57–1.05)	0.099	0.98 (0.69–1.41)	0.93	EMT; GPCR signaling; double–strand break repair
PRAD_M1	6.42 (0.78–52.60)	0.049	1.80 (1.16–2.79)	0.0081	Calcium ion homeostasis; muscle contraction; mesoderm morphogenesis
PRAD_M2	0.42 (0.08–2.19)	0.29	0.52 (0.33–0.81)	0.0032	Calcium ion homeostasis; cell-cell adhesion
THCA_M1	1.21 (0.45–3.23)	0.7	0.55 (0.31–0.99)	0.041	Stem cell proliferation
UCEC_M3	1.81 (1.15–2.84)	0.0087	1.66 (1.12–2.47)	0.01	Type I interferon production; DNA duplex unwinding

*OS, overall survival; PFS, progression-free survival; HR, hazard ratio; P, Log-rank test p-value*.

**Figure 4:**
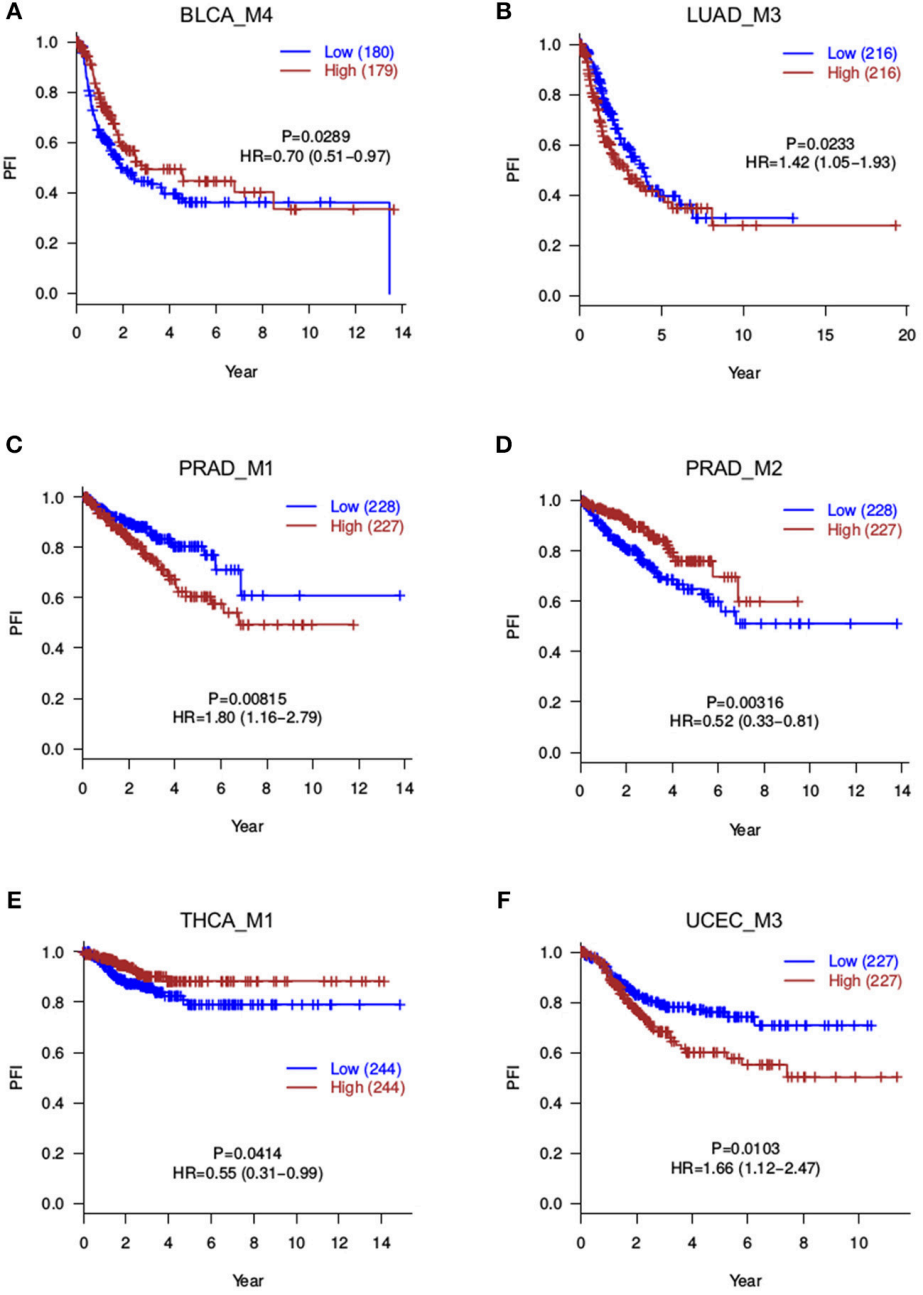
Progression-free survival analyses for splicing modules. Red curves are with a high module score, and blue curves with a low module score. **(A)** BLCA_M4; **(B)** LUAD_M3; **(C)** PRAD_M1; **(D)** PRAD_M2; **(E)** THCA_M1; **(F)** UCEC_M3.

Besides overall survival (OS) that reflects a long-term prognosis, it is often of interest to evaluate short-term effects on disease progression. Therefore, to further capture more prognosis-related modules, we also tested the correlation between module scores and progression-free intervals (PFI). This analysis returned 8 significant modules (*P* ≤ 0.05), namely, BLCA_M1, BLCA_M2, BLCA_M4, LUAD_M3, PRAD_M1, PRAD_M2, THCA_M1, and UCEC_M3 (Table 2, Figure 4). Note that BLCA_M4 is also marginally significant in OS analysis (*P* = 0.076, HR = 0.75 with a confidence interval of 0.54–1.03), while PRAD_M2 (OS *P* = 0.29) and THCA_M1 (OS *P* = 0.7) are only significant in PFI analysis. Five modules are strictly significant in both the OS and PFI settings (BLCA_M1, BLCA_M2, LUAD_M3, PRAD_M1, and UCEC_M3), and interestingly, their HR ratios in these two settings are in a similar trend, either both reducing or both increasing malignancy risks. BLCA_M1 lowers both the death risk (0.57, 0.41–0.78) and the disease progression risk (0.67, 0.48–0.91); BLCA_M2 increases both the death risk (1.78, 1.29–2.48) and the progression risk (1.42, 1.04–1.95); LUAD_M3 also increases both risks (1.86, 1.34–2.58 and 1.42, 1.05–1.93, respectively); PRAD_M1 also increases both risks (6.42, 0.78–52.60 and 1.80, 1.16–2.79, respectively); UCEC_M3 similarly increases both risks (1.81, 1.15–2.84 and 1.66, 1.12–2.47, respectively). These strongly indicate the consistency of splicing modules as potential prognosis biomarkers, suggesting underlying functional involvement of these modules in their corresponding cancer types.

### Cancer Splicing Modules Are Enriched for Critical Biological Functions

The above analyses yielded 15 modules with potential prognosis relevance (Table 2). To characterize the functional properties of each splicing module, GO enrichment analysis was performed on the 15 modules. The major functional implications of each module were manually examined and curated from the enrichment results (Table 2). Since nearly all genes transcribed in the genome, including many long non-coding genes, underwent alternative splicing, typically very few events could drive strong functional changes, and the majority of alternative splicing events at most function as weaker modifiers. Surprisingly, we found that the 15 modules, when compared to the splicing events catalog, showed very strong enrichment of important biological functions, such as stem cell proliferation and epithelial-mesenchymal transition (EMT), (BLCA_M1, LIHC_M2, LUSC_M1, THCA_M1, THCA_M2, UCEC_M2), cell cycle control (BRCA_M1, BRCA_M2, COAD_M4, KICH_M1, STAD_M1), DNA repair or regulation (COAD_M3, ESCA_M2, HNSC_M3, KICH_M1, LUAD_M3, LUSC_M3, UCEC_M3), developmental cell growth (BRCA_M4, COAD_M1, LIHC_M1, LIHC_M3, READ_M1, READ_M2, STAD_M2, STAD_M3), receptor or kinase signaling pathways (BLCA_M4, HNSC_M1, KICH_M1, KIRC_M2, KIRP_M1, KIRP_M2, LIHC_M1, LIHC_M2, LUAD_M1, LUAD_M2, LUSC_M1, LUSC_M3, READ_M2), VEGF-mediated vessel development (LUAD_M1, LUSC_M1) (Table 2). Among these major functions, EMT is required for cancer invasion and metastasis, which is closely related to cancer mortalities and prognosis (Singh and Settleman, 2010). The important EMT-related gene modulated in BLCA and LIHC is FGFR2, which regulates mesenchymal condensation in BLCA (Chaffer et al., 2006). Targeting FGFR signaling through splicing factors might expand the current toolkits (Touat et al., 2015). Vessel development controlled by VEGF signaling is another pathway directly involved in cancer metastasis and patient survival (Stacker et al., 2002; Su et al., 2006). Both VEGFA and its receptor FLT4 (VEGFR-3) were altered during splicing in lung cancers LUAD and LUSC, which might modulate angiogenesis through splicing control. In summary, these suggest that the splicing network-based module identification approach taken in this study was powerful enough to extract the few critically functional events from a much larger splicing background.

### Splicing Modules Across Cancer Types Reveal Pan-Cancer Signatures

**Figure 5:**
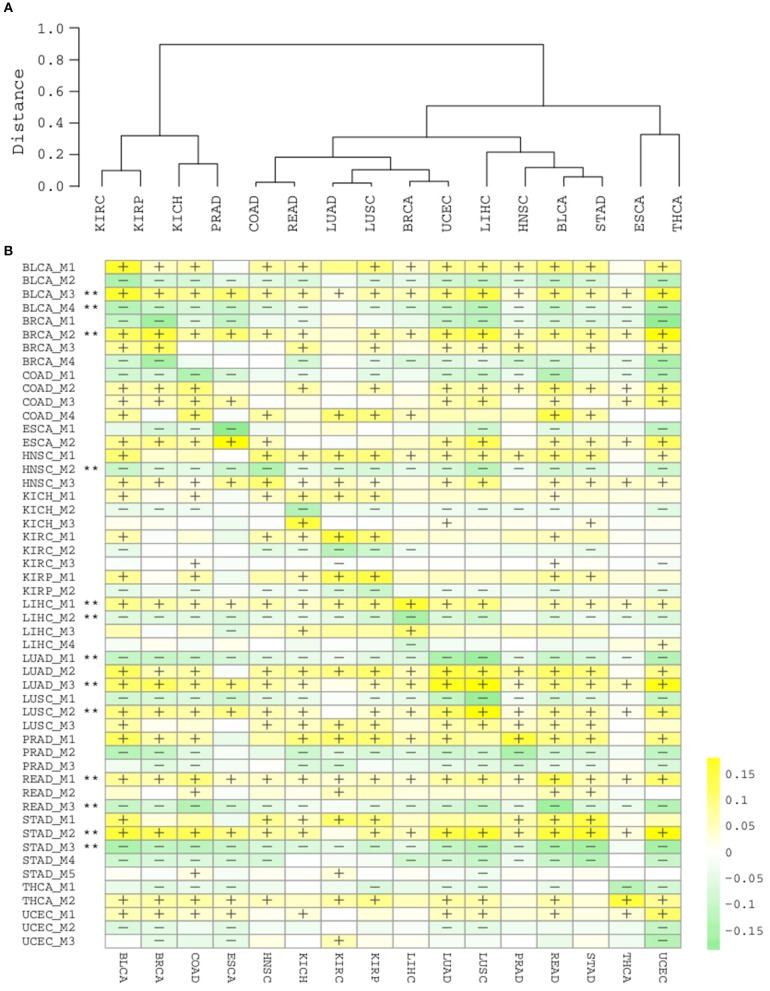
**(A)** Hierarchical clustering of cancer types by 51 splicing modules. **(B)** Heatmap of module scores in each cancer type. “+” and “–” respectively denote that for ≥80% samples in the cancer type are with a higher and lower module score than normal samples. “**” denotes the module are consistently “+” or “–“ in ≥15/16 cancer types.

Having obtained those functionally coherent modules, we next asked whether it would be helpful to explore the pan-cancer landscape at the module level. Hierarchical clustering of cancer types with the 51 modules revealed a clear pattern that is closely related to tissue origins (Figure 5A). Lung cancers (LUAD, LUSC), colon cancers (COAD, READ), gynecological cancers (BRCA, UCEC), kidney, and prostate cancers (KIRC, KIRP, KICH, PRAD) each are clustered in a tissue origin manner. This is actually consistent with a recent pan-cancer analysis using multi-platform integrative clustering (Hoadley et al., 2018), suggesting that splicing events can also be useful for cancer classification and subtyping.

Due to the intra-type and between-type heterogeneity of cancers, it is important to know which of the splicing modules are shared by multiple cancer types and which modulesare restricted to one or few cancer types. We summarized the scores for each module in the cancer samples and categorized them by cancer type (Figure 5B). The diagonal line here reflects the score of modules in their corresponding cancer types, while the off-diagonal regions depicts their pan-cancer potential. Although a few modules from kidney and liver cancers show a strong cancer type specificity, and largely not perturbed in other types (KICH_M3, LIHC_M3, LIHC_M4), many other modules display strong pan-cancer perturbation patterns, suggesting their wider involvement in most cancer types. With a strict criteria (perturbation found in at least 15/16 cancer types), we found that 13/51 modules are highly common across different cancer types (marked with ^**^, Figure 5B), again suggesting that the modules identified with splicing network analysis are highly informative and important.

## Discussion

Although AS has been identified and studied for many years, the full regulation pattern of these many AS events within and across cancer types are still not completely understood. Previous studies have taken advantage of single-event analyses and linked splicing to splicing factors as well as the cis-elements. Very recently, an interesting study sets out to determine the involved of spliceosome RNAs in cancer-specific AS regulation (Dvinge et al., 2018).

In this study, we have taken a novel approach that emphasizes the inter-event correlations and uncovers the modularized perturbation of splicing events in cancers. Previous studies have not emphasized the modularized control of splicing events, which according to our study is quite important. Indeed, a relatively small number of functionally important and prognosis-relevant modules have been successfully identified, with some of them being common across cancer types and others being more specific to one or few cancer types, indicating that our approach is both powerful and useful.

To focus on the more typical AS classes, we have not considered alternative promoters in this study, as their regulation are more relevant to transcriptional factors, enhancers or even epigenetic modifications (Maunakea et al., 2010; Kowalczyk et al., 2012). Nonetheless, it would be interesting to investigate the possibility of combining transcriptional events and splicing events in the future, as co-transcriptional splicing has already been proposed and supported by various studies. This might serve as a plausible framework for those interactions (Lee and Rio, 2015).

## Data Availability

The datasets used for this study are publicly accessible, and are also available by contacting the corresponding authors.

## Author Contributions

WM, TZ, and ZF: conceived and oversaw the study, wrote the manuscript. YD, SL, RD, and YZ: performed the data analysis. NS, SA, SC, and AW: assisted in data analysis and interpretation.

### Conflict of Interest Statement

Author YZ was employed by the company Medcurius Co. The remaining authors declare that the research was conducted in the absence of any commercial or financial relationships that could be construed as a potential conflict of interest.

## Supplementary Material

Figure S1 Comparison of Pearson and Kendall correlation coefficients on network and module detection. NMI (normalized mutual information) and ARI (adjusted Rand index) are used to evaluate consistency.

Figure S2 Consistency between average and median module scores on survival analyses. **(Left)** OS analysis. **(Right)** PFI analysis. Each circle or “+” refers to a module.

Table S1 Detailed splicing events within each of the 51 splicing modules. Each event is named as “gene::eventID::eventType”.
