# Corrigendum: Modularized Perturbation of Alternative Splicing Across Human Cancers

**DOI:** 10.3389/fgene.2019.00969

**Published:** 2019-10-03

**Authors:** Yabing Du, Shoumiao Li, Ranran Du, Ni Shi, Seiji Arai, Sai Chen, Aijie Wang, Yu Zhang, Zhaoyuan Fang, Tengfei Zhang, Wang Ma

**Affiliations:** ^1^Department of Oncology, The First Affiliated Hospital of Zhengzhou University, Zhengzhou, China; ^2^Department of Surgery, Anyang Tumor Hospital, Anyang, China; ^3^Institute of Medical Information, Chinese Academy of Medical Sciences, Peking Union Medical College, Beijing, China; ^4^Comprehensive Cancer Center, Ohio State University, Columbus, OH, United States; ^5^Department of Hematology and Oncology, Beth Israel Deaconess Medical Center, Harvard Medical School, Boston, MA, United States; ^6^Department of Urology, Gunma University Graduate School of Medicine, Maebashi, Japan; ^7^Department of Clinical Medicine, Xinxiang Medical University, Xinxiang, China; ^8^Bioinformatics Group, Medcurius Co., Zhejiang, China; ^9^Shanghai Institutes for Biological Sciences, Chinese Academy of Science, Shanghai, China; ^10^Medical College, Henan Polytechnic University, Jiaozuo, China

**Keywords:** alternative splicing, splicing network, splicing modules, cancer splicing, prognosis

In the original article, there was an error. The “Correspondence emails” are listed in the incorrect order.

A correction has been made to the ***Correspondence***.

*Correspondence:

Wang Ma

doctormawang@126.com

Tengfei Zhang

fcczhangtf@zzu.edu.cn

Zhaoyuan Fang

fangzhaoyuan@sibs.ac.cn

The authors apologize for this error and state that this does not change the scientific conclusions of the article in any way. The original article has been updated.

