# Supplementary figures and images for: Modularized Perturbation of Alternative Splicing Across Human Cancers
Source: Front Genet. 2019 Apr 3;10:246. doi: 10.3389/fgene.2019.00246 (PMC6463002; doi:10.3389/fgene.2019.00246)

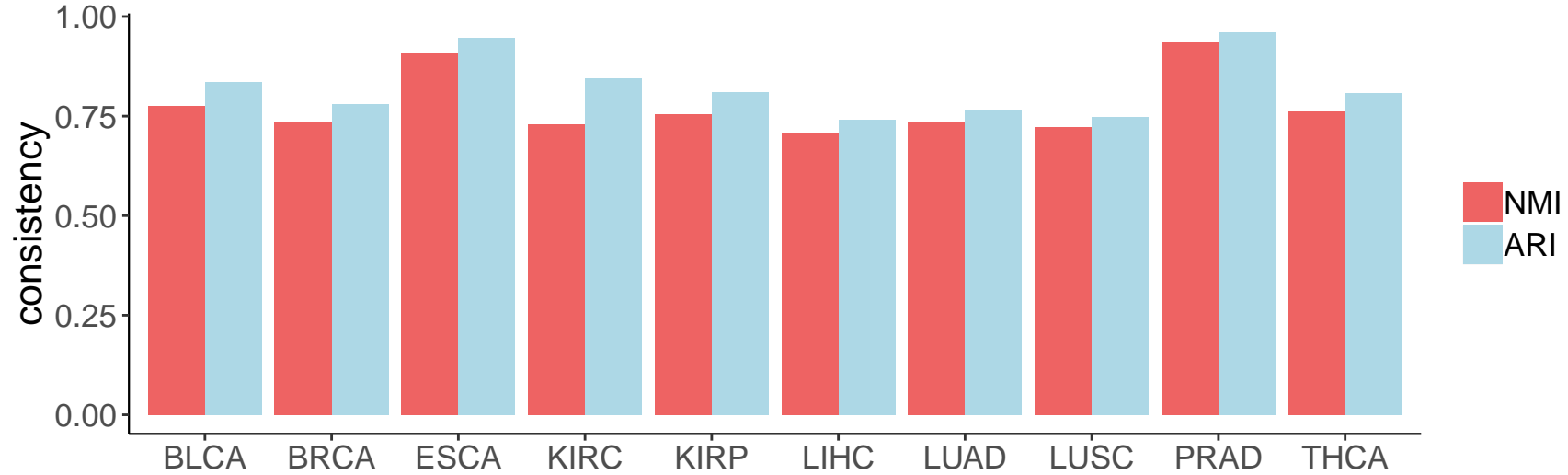

Supplement: Figure S1 — Comparison of Pearson and Kendall correlation coefficients on network and module detection. NMI (normalized mutual information) and ARI (adjusted Rand index) are used to evaluate consistency. [file Image_1.pdf]

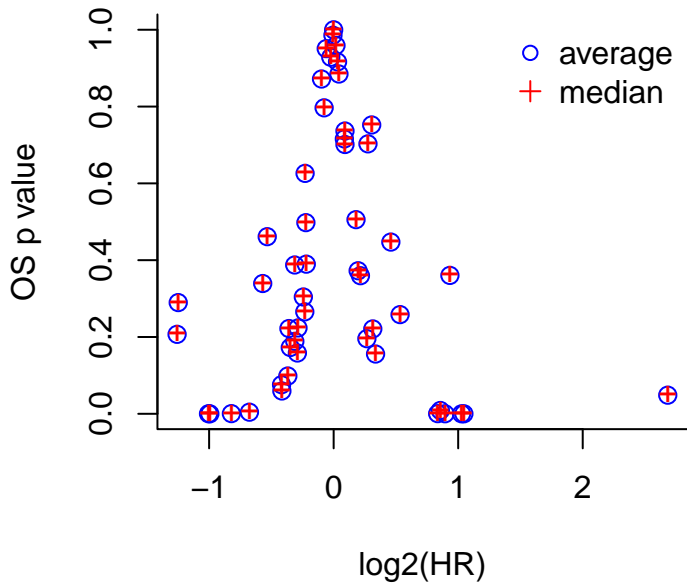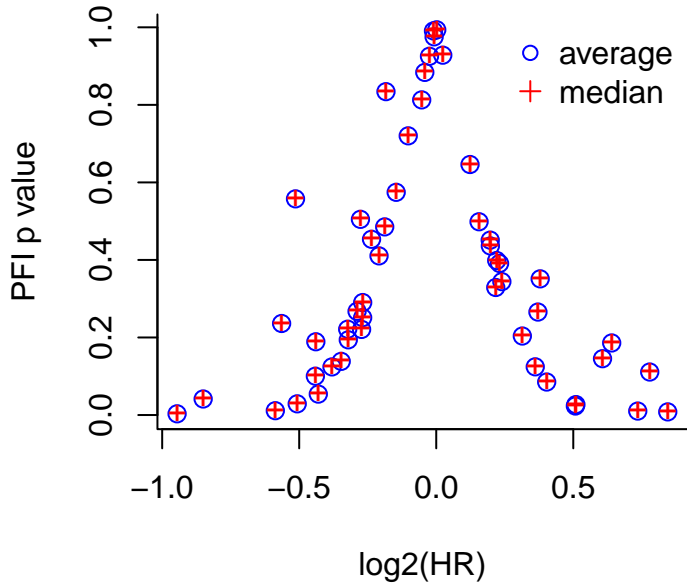

Supplement: Figure S2 — Consistency between average and median module scores on survival analyses. (Left) OS analysis. (Right) PFI analysis. Each circle or “+” refers to a module. [file Image_2.pdf]
